# Assessment of the progression of kidney renal clear cell carcinoma using transcriptional profiles revealed new cancer subtypes with variable prognosis

**DOI:** 10.3389/fgene.2023.1291043

**Published:** 2023-11-24

**Authors:** Michelle Livesey, Nasr Eshibona, Hocine Bendou

**Affiliations:** ^1^ SAMRC Bioinformatics Unit, South African National Bioinformatics Institute, University of the Western Cape, Cape Town, South Africa; ^2^ Computational Biology Division, Department of Integrative Biomedical Sciences, Faculty of Health Sciences, University of Cape Town, Cape Town, South Africa

**Keywords:** kidney renal clear cell carcinoma, normalization, cancer progression, subtypes, prognosis, gene signature

## Abstract

**Background:** Kidney renal clear cell carcinoma is the most prevalent subtype of renal cell carcinoma encompassing a heterogeneous group of malignancies. Accurate subtype identification and an understanding of the variables influencing prognosis are critical for personalized treatment, but currently limited. To facilitate the sub-classification of KIRC patients and improve prognosis, this study implemented a normalization method to track cancer progression by detecting the accumulation of genetic changes that occur throughout the multi-stage of cancer development.

**Objective:** To reveal KIRC patients with different progression based on gene expression profiles using a normalization method. The aim is to refine molecular subtyping of KIRC patients associated with survival outcomes.

**Methods:** RNA-sequenced gene expression of eighty-two KIRC patients were downloaded from UCSC Xena database. Advanced-stage samples were normalized with early-stage to account for differences in the multi-stage cancer progression’s heterogeneity. Hierarchical clustering was performed to reveal clusters that progress differently. Two techniques were applied to screen for significant genes within the clusters. First, differentially expressed genes (DEGs) were discovered by Limma, thereafter, an optimal gene subset was selected using Recursive Feature Elimination (RFE). The gene subset was subjected to Random Forest Classifier to evaluate the cluster prediction performance. Genes strongly associated with survival were identified utilizing Cox regression analysis*.* The model’s accuracy was assessed with Kaplan-Meier (K-M)*.* Finally, a Gene ontology and Kyoto Encyclopedia of Genes and Genomes enrichment analyses were performed.

**Results:** Three clusters were revealed and categorized based on patients’ overall survival into short, intermediate, and long. A total of 231 DEGs were discovered of which RFE selected 48 genes. Random Forest Classifier revealed a 100% cluster prediction performance of the genes. Five genes were identified with significant diagnostic capacity. The downregulation of genes *SALL4* and *KRT15* were associated with favorable prognosis, while the upregulation of genes *OSBPL11*, *SPATA18*, and *TAL2* were associated with favorable prognosis.

**Conclusion:** The normalization method based on tumour progression from early to late stages of cancer development revealed the heterogeneity of KIRC and identified three potential new subtypes with different prognoses. This could be of great importance for the development of new targeted therapies for each subtype.

## 1 Introduction

Multiple different forms of kidney tumors make up the complex disease known as kidney cancer (Hu et al., 2019). Renal cell carcinoma (RCC) is a heterogeneous group of kidney parenchyma tumors that can be further divided into histologically defined subtypes (Znaor et al., 2015; Casuscelli et al., 2017; Xiong et al., 2022). The different subtypes have undergone multiple revisions in the past two decades, due to advancements in the morphological as well as molecular characterization of renal tumors (Kovacs et al., 1997; Lopez-Beltran et al., 2006; Srigley et al., 2013; Moch et al., 2016; Udager and Mehra, 2016).

The recent discoveries in renal tumor transcriptome profiling studies have had a substantial influence in the field of genomics as a category for “molecularly defined renal carcinomas” has been introduced by the World Health Organization 2022 classification of urinary and male genital tumors (5th edition) (Trpkov et al., 2021a; 2021b; Mohanty et al., 2023). These studies have significantly improved our understanding of RCC, however, effective diagnostic and therapeutic approaches have yet to be achieved (Caliskan et al., 2020). Additionally, these studies revealed the high molecular heterogeneity of these tumors, necessitating further sub-classification.

In this study, the most prevalent and aggressive subtype Kidney renal clear cell carcinoma (KIRC) was investigated as it accounts for 80%–90% of the total number of RCC patients (Wang Q. et al., 2019). Patients with KIRC are associated with a high mortality rate and poor clinical outcomes (Gray and Harris, 2019; Puzanov, 2022). Also, there are limited therapeutic options available; surgery is the primary option since KIRC is resistant to radiotherapy and chemotherapy (Yin et al., 2019). The resistance to treatment may be due to the heterogeneity of these tumors. Therefore, an accurate assessment of the heterogeneity of these tumors is crucial to identify subtypes of patients that can benefit from targeted therapy. This can be achieved by investigating the underlying molecular mechanisms and progression of KIRC, which are currently not fully understood (You et al., 2021).

To track cancer progression we implemented a recently established normalization method, which also has the potential to facilitate the sub-classification of KIRC (Livesey et al., 2023). The normalized gene expression reveals how cancer progresses by detecting the accumulated genetic changes that emerge from early-stages of cancer development to advanced-stages. The application of the normalization method and hierarchical clustering will allow for the identification of clusters (subtypes) that progress differently.

This study aims to reveal KIRC patients with different progression (subtypes) and establish a genotype-phenotype link to the identified clusters. In this study, the genotype-phenotype relationship to the distinct clusters was defined by the average overall survival (OS) of the KIRC patient samples. Prognostic gene signatures were identified that differentiate between the different survival clusters and have the potential to function as prognostic biomarkers that can facilitate the prognosis and monitoring of KIRC. Therefore, the study advances knowledge of the transcriptional landscape of KIRC patients with an emphasis on cancer progression.

## 2 Materials and methods

### 2.1 Data acquisition and processing

The RNA-Sequencing (RNA-Seq) gene expression profiles of KIRC were downloaded from the UCSC Xena database using cancer-specific data from The Cancer Genome Atlas cohort, from the Genomic Data Commons (GDC-TCGA) (Goldman et al., 2020). A total of eighty-two advanced-stage cancer samples, along with a matched number of randomly selected early-stage samples were extracted. The accompanying metadata included the corresponding patient phenotypic and survival profiles.

The gene expression profile of each patient was organized in a gene-by-sample genomic matrix. The cancer datasets consisted of 60,483 unique Ensembl identifiers (ENSG) (Aken et al., 2016), quantified as log_2_(x+1), where x represents the count of reads mapped to a specific genomic location in the human reference genome (GRCh38.p2, gencode release 22). Ensembl BioMart (GRCh38.p13, Ensembl 104 May 2021) (Smedley et al., 2015) was utilized to retrieve a total of 19,556 ENSG identifiers that were annotated with a protein-coding biotype. Hence, 40,927 (67, 7%) non-coding entries were eliminated. For further analysis, the 19,556 protein-coding gene expressions were converted to counts.

### 2.2 Data normalization

The normalization method that tracked cancer progression and corrected for multiple cancers (Livesey et al., 2023) was modified to investigate a cancer type. The normalization method involves calculating the quotient of advanced-stage gene expression and early-stage gene expression.

#### 2.2.1 Tracking cancer progression

A normalization method was implemented to capture the heterogeneity between cancerous tumors by detecting their molecular differences in progression from early to late-stages of tumor development using gene expression by RNA-Seq. As a result, the method exposes the accumulated genetic changes that occur throughout the multi-stage of cancer development. To track the development of cancer, the gene expression profiles of both early-stage and late-stage cancer samples were required. Thus, the gene-by-sample matrix of KIRC was used to create two distinct matrices; early-stage (E) and advanced-stage (A) gene expression as follows:

E, s x r matrix for early-stage gene expression and,

A, s x q matrix for advanced-stage gene expression.

The early-stage and advanced-stage gene expression matrices are represented by E and A, respectively. Where r and q corresponds to the number of cancer samples in early-stage and advanced-stage, and s the number of protein-coding genes represented with raw count gene expression value.

The early-stage patient profiles do not match the same patient profiles in the late-stages. Thus, the initial approach to calculating the normalized dataset involves generating a mean normalized expression, or “m_*i*_”, for gene *i* in the early-stage dataset*.* The sum of early-stage gene *i* for all early-stage cancer *k* samples was calculated, as shown in Eq 1. The average early-stage expression vector of gene *i* produced by this equation offers a more accurate representation of the early-stage expression of a particular gene.

$$m_{i}=\frac{1}{r}\sum_{k=1}^{r}E_{i,k}$$ (eq 1)

$$L_{i}=\ln\left(\frac{A}{m_{i}}\right)$$ (eq 2)

Finally, the gene expression matrix that represents cancer progression, L was calculated as demonstrated in Eq 2. Matrix L contains normalized counts of the quotients of advanced-stage (dividend) and the mean gene expression of early-stage cancer samples (divisor). Therefore, the normalized gene expression represents the continuously changing cellular transcriptome, allowing for an efficient and comprehensive description of gene expression profiles.

### 2.3 Hierarchical clustering

The clustering of cancer samples is the most fundamental strategy to identify groups of samples that progressed differently in gene expression patterns. This approach may result in the identification of novel cancer clusters (subtypes) within a cancer type. Therefore, the normalized gene expression profiles of the KIRC cancer samples were subjected to hierarchical clustering analysis, to reveal the grouping of cancer samples.

The clusters of cancer samples were created by hierarchical clustering, using the cosine distance between the gene expression profiles and Ward’s method for agglomeration (Ward, 1963; Jaskowiak et al., 2014). The optimal number of clusters was determined using the *find_k* function as part of the dendextend R package (version 1.17.1), which calculates k using maximal average silhouette widths (Rousseeuw, 1987). Finally, the dendrograms were split into k groups to assign samples to a cluster.

### 2.4 Feature analysis

#### 2.4.1 Differential gene expression

Limma package in R (version 3.54.2) (Ritchie et al., 2015) was used to screen for differentially expressed genes (DEGs), by applying an empirical Bayesian approach to evaluate for differences in gene expression profiles between the identified clusters. The *decideTests* (Law et al., 2016) function assigned binary values (0: not detected, 1: upregulated, and −1: downregulated) to the genes, to identify and extract genes that differentiate between the altered (up or down) gene expression. Significant DEGs were defined as those with a Benjamini–Hochberg (BH) adjusted *p*-value <0.05 and log2-fold change (LFC) ≥ 0.5 or ≤ −0.5.

#### 2.4.2 Marker gene selection using machine learning

Recursive Feature Elimination (RFE) algorithm was implemented to identify key genes playing a role in the classification of the identified KIRC clusters (subtypes), using the Scikit-learn python package (Pedregosa et al., 2011). RFE with a linear kernel support vector machine (SVM) was utilized to find optimal genes that predict the cancer clusters. The k-fold cross-validation procedure, with a value of K set to 10, was repeated 3 times.

The model was built with all identified DEGs and in several iterations eliminates a single gene deemed least important for segregating the identified clusters (Guyon et al., 2002). The model is rebuilt, and the new gene subset are evaluated based on their classification performance. Hence, the genes are ranked according to their relevance. In this study, the final gene subset was selected based on the highest classification accuracy by linear SVM with *C* set to 5. The final gene subset was further subjected to principal component analysis (PCA) using the R packages FactoMineR (version 2.8) (Lê et al., 2008) and factoextra (version 1.0.7) (Kassambara and Mundt, 2020).

### 2.5 Predictive and validation of marker genes

The performance of the RFE selected gene subset was validated using Random Forest (RF) classifier with a “test-train split ()” class to split the data into train and test sets with a ratio of 75: 25. The performance of the RF classifier was measured using accuracy, precision, and recall score as the performance metrics. All machine learning implementations were run in Anaconda environment based on python programming language and Scikit-learn package (Pedregosa et al., 2011).

### 2.6 Survival analysis

The gene subset selected by RFE was subjected to a Cox regression model based on the Lasso algorithm of the glmnet R package (version 4.1-7), to further understand the relative importance of the gene subset (Friedman et al., 2010; Simon et al., 2011; Tibshirani et al., 2012). The model reduces the total number of the gene subset and identifies the genes with the most significant impact on a patient’s survival. This step assigned a regression coefficient value to the given gene that is multiplied by the corresponding gene’s expression and results in a prognostic risk score for each patient. The patient scores were used to calculate a median risk score. Each patient was assigned a status value of 0 or 1 based on whether the patient’s score was higher or lower than the median risk score. The patient status information was used to generate Kaplan-Meier (K-M) estimates for OS. The K–M curves were constructed using the *ggsurvplot* function from the survminer R package (version 0.4.9).

### 2.7 One-way ANOVA

A one-way analysis of variance (ANOVA) was performed to compare the mean gene expression of the prognostic genes discovered by Cox regression analysis between the identified clusters. Statistical analysis was conducted with the stats R package (version 4.2.2). Following the application of ANOVA, Tukey’s *post hoc* test for pairwise comparisons was applied (Tukey, 1949). The null hypothesis (H0) of equal mean between the clusters was rejected if the *p*-value < 0.05; H1: the cluster means are significantly different from one another.

### 2.8 Enrichment

The list of DEGs were subjected to functional annotations of Gene ontology (GO) (Ashburner et al., 2000), with an adjusted *p*-value < 0.05 determined as a cut-off criterion for significant enrichment. Additionally, the 48 RFE gene subset were subjected to Kyoto Encyclopedia of Genes and Genomes (KEGG) pathways enrichment, with the threshold for significant enrichment established as *p*-value <0.05. The enrichment analysis was performed utilizing the clusterProfiler R package (version 4.6.2) (Yu et al., 2012).

## 3 Results

### 3.1 Cancer clusters detection with normalized expression

The gene expression profiles of eighty-two advanced-stage KIRC samples were normalized with early-stage cancer samples to consider the heterogeneity differences that occur in the multi-stage cancer progression.

**FIGURE 1:**
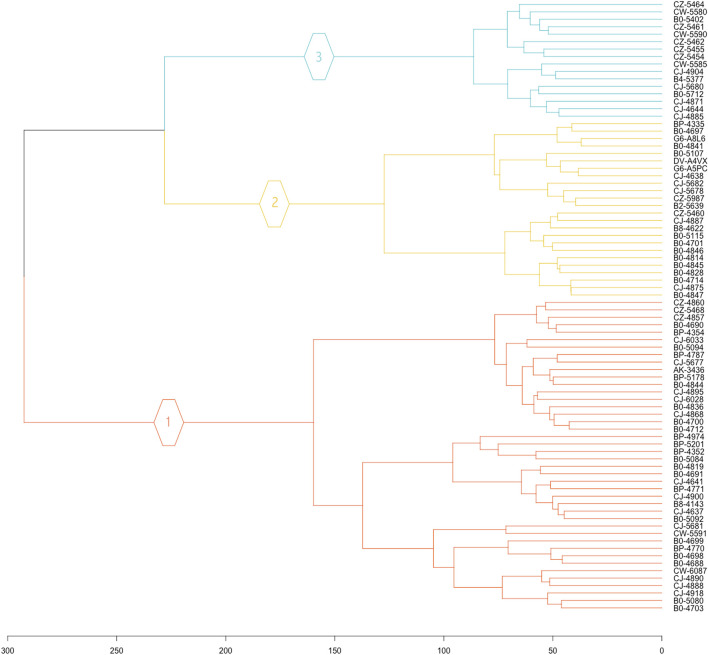
Hierarchical clustering dendrogram of KIRC patient. The 19,556 normalized gene expression profiles of the eighty-two KIRC cancer samples were subjected to clustering analysis, to reveal the grouping of cancer samples.

In this study, all 19,556 normalized protein-coding genes were subjected to clustering. The clusters are visually represented in a hierarchical tree called a dendrogram. The clustering of all eighty-two KIRC samples revealed three unique KIRC progression patterns based on gene expression profiles (Figure 1).

Three unique cancer clusters (subtypes) as Clusters 1, 2, and 3 were identified and encompass a total of 42, 24, and 16 KIRC patient samples, respectively. These three molecularly identified clusters were further correlated with the patients’ average overall survival to reflect its genotype-phenotype relationship. Cluster 1 showed the lowest average OS of 864.43 days, Cluster 2 displayed an average OS of 1076.38, and Cluster 3 had the highest average OS of 1522.31 days. Therefore, these Clusters were categorized as Short (SS), Intermediate (IS), and Long Survival (LS) (Table 1).

**TABLE 1:** The number of patient samples stratified by hierarchical clustering. The average overall survival of all patients within a cluster was calculated and further categorized into Short (SS), Intermediate (IS), and Long Survival (LS).

Cluster	Average survival (days)	Survival time	Risk subcategory	Samples
1	864.43	Short	SS	42
2	1076.38	Intermediate	IS	24
3	1522.31	Long	LS	16
Total				82

### 3.2 Differential gene expression analysis

In the differential gene expression (DGE) analysis, a total of 19,556 protein-coding genes were evaluated for DEGs to distinguish between SS, IS, and LS. A pairwise comparison approach between the gene expression profiles of IS and SS, LS and SS, and LS and IS were used, and only the genes with an adjusted *p*-value <0.05 and LFC ≥0.5 or ≤ −0.5 between all three pairwise comparisons were used for further analysis. Thus, a total of 231 DEGs were discovered.

Considering only the DEGs that were significant between all three pairwise comparisons, a total of 47 genes were identified as upregulated, when IS was compared to SS, whereas 184 genes were found to be downregulated. While 159 genes were upregulated, and 72 genes were downregulated in the comparison of LS and SS. Finally, the comparison of LS and IS, identified 221 and 10 genes as upregulated and downregulated, respectively.

### 3.3 Selection of optimal gene subset

**FIGURE 2:**
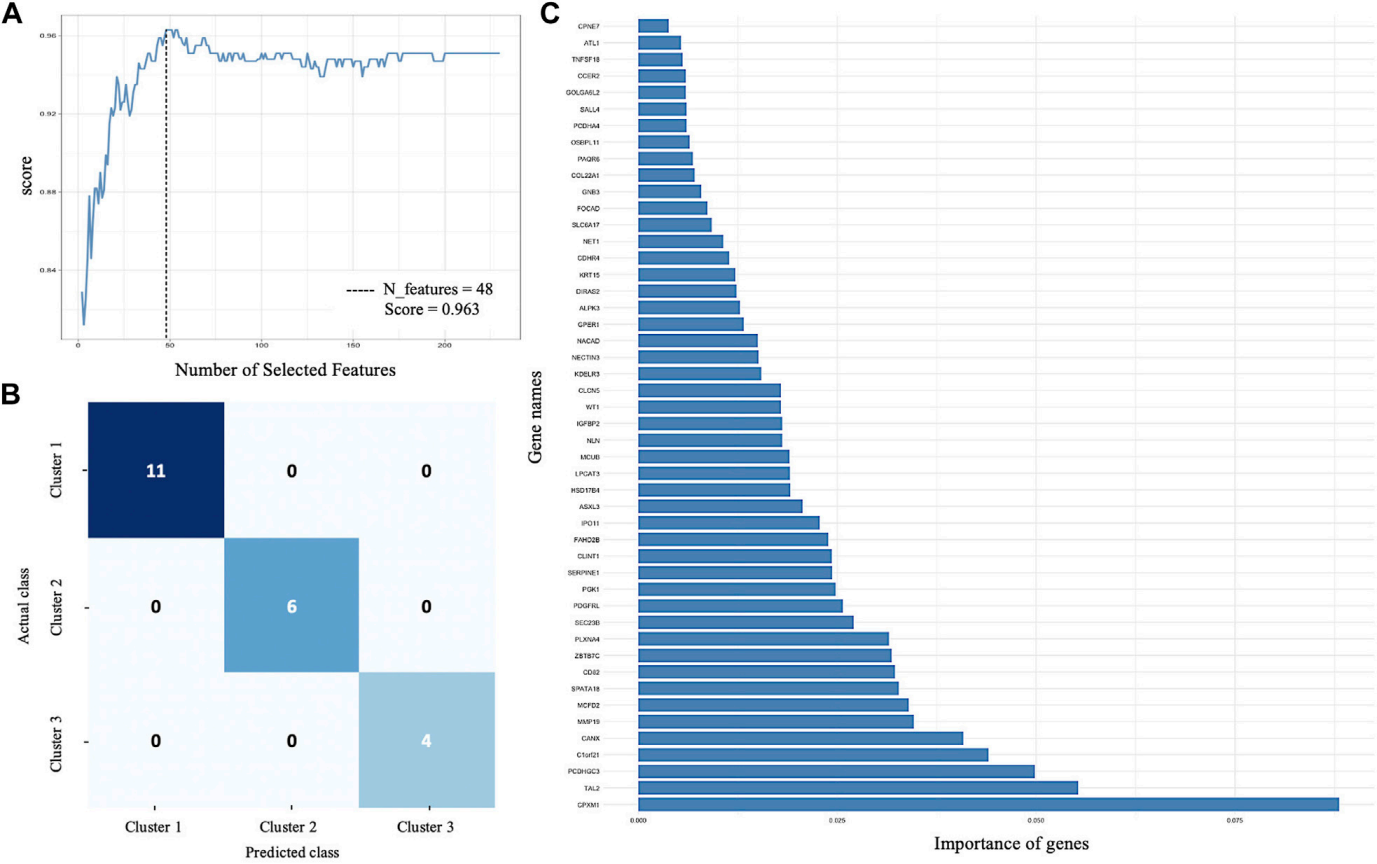
Supervised machine learning. **(A)** Recursive feature elimination selected 48 genes with the highest performance score of 0.963. **(B)** Confusion matrix that defines the performance of RF classifier. Each row and columns represent the instances in an actual and predicted class, respectively. **(C)** The importance of each gene for RF classifier prediction.

All 231 DEGs identified between SS, IS, and LS KIRC patients were screened by the RFE algorithm. The optimal gene subset is defined by the best combination of genes that has candidate characteristics of classification and prognosis. This also refers to the performance of the RFE and is quantified by the feature importance score. In this study, the optimal gene subset of 48 genes (Supplementary Table S1) with the highest performance score of 0.963 was selected for further analysis (Figure 2A).

#### 3.3.1 Validation of optimal RFE gene subset

An RF classifier model was constructed to evaluate the classification power of the 48 RFE gene subset for SS, IS, and LS. A tenfold cross-validation on a forest model in the training phase (75% of the samples) and testing phase (25% of the samples) was computed. The Random Forest classification yielded an accuracy score of 100%, a precision of 100%, and a recall of 100%.

A confusion matrix that defines the performance of the classification algorithm is presented in Figure 2B. The importance of each gene for risk subcategory prediction to the RF classifier model is presented in Figure 2C.

A PCA model was built to determine the heterogeneity in gene expression between the SS, IS, and LS risk subcategories. The PCA assessed and identified the key sources of variance, allowing samples to be grouped based on similar and different gene expression profiles.

**FIGURE 3:**
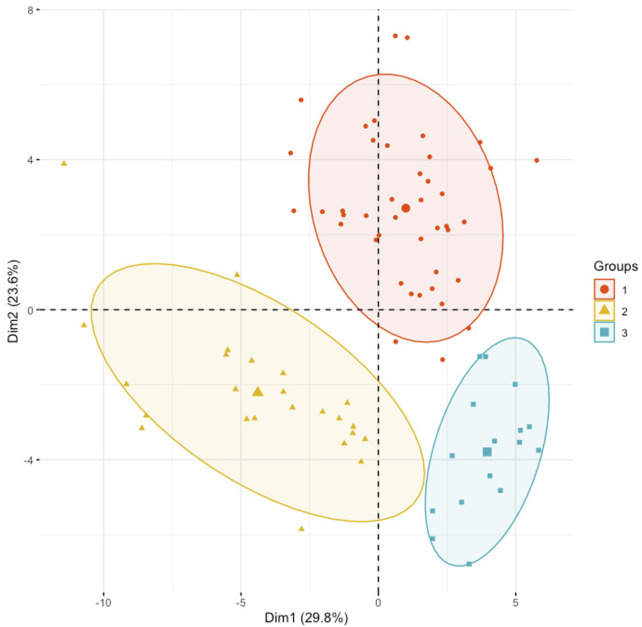
Principal component analysis using the normalized gene expression profiles of the 48 RFE gene subset. KIRC samples were stratified according to the initial hierarchical clustering analysis.

Dim 1 represented 29.8% of the overall variance, whereas Dim 2 represented 23.6% (Figure 3). A clear segregation between KIRC patient samples can be observed to distinguish between the three risk subcategories.

**FIGURE 4:**
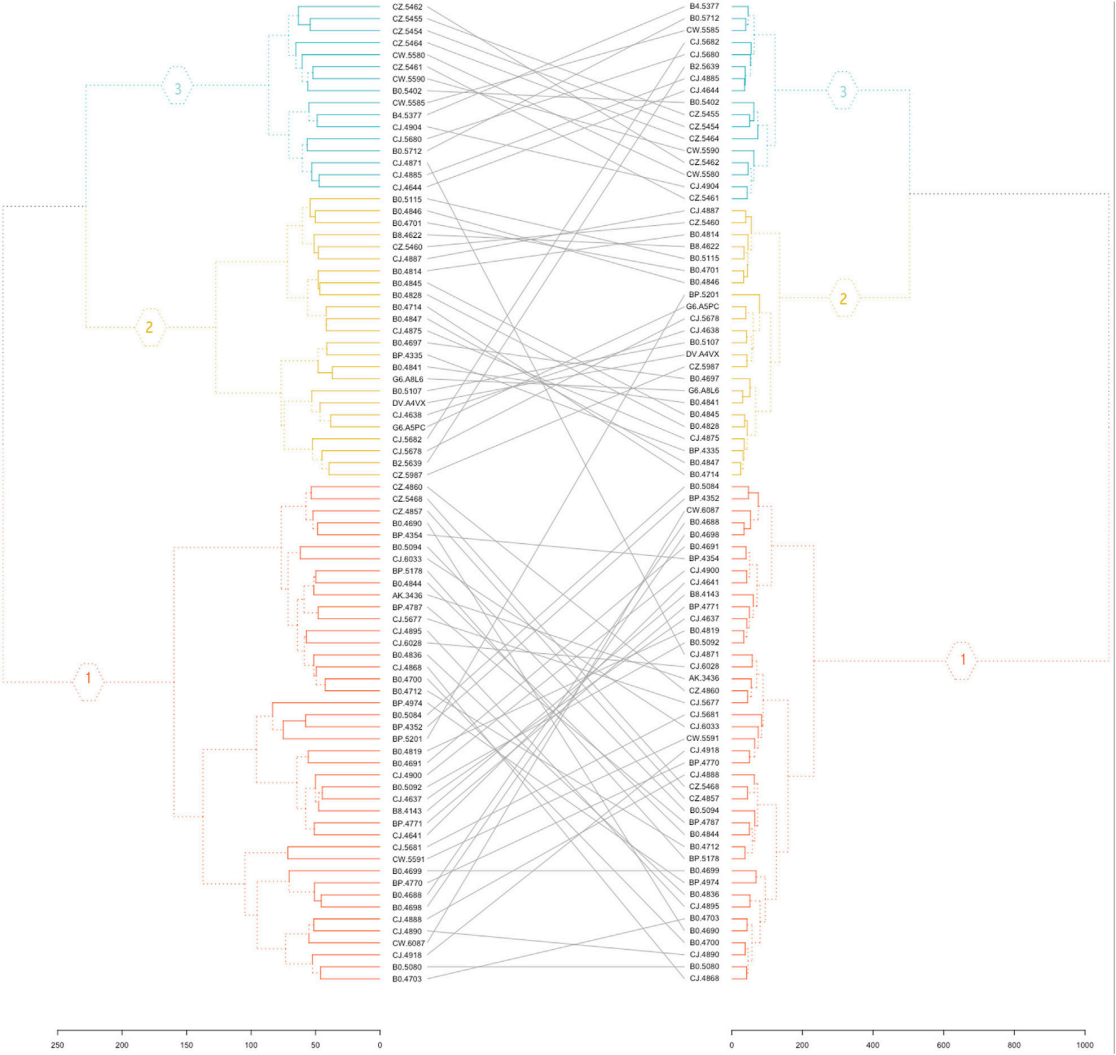
Tanglegram. The initial hierarchical clustering of 19,556 protein-coding genes (left) and clustering analysis of the 48 RFE gene subset (right).

To further compare the initial clustering analysis of protein-coding genes to the clustering of the selected 48 RFE gene subset, a hierarchical clustering was performed with the normalized gene expression of the 48 RFE gene subset of the eighty-two KIRC cancer samples. The correspondence between the two hierarchical clusters is represented by a tanglegram (Figure 4). It can be observed that only four samples were assigned to a different cluster (risk subcategory) with the reduced gene subset (Figure 4).

### 3.4 Identification of prognostic genes

Five prognostic genes were identified and linked with KIRC patient survival by univariate Cox regression analysis between the 48 RFE gene subset and patient survival data. The prognostic genes were detected utilizing the LASSO algorithm, which assigns non-zero, positive, or negative coefficients. Two of the five genes had positive coefficients, while three genes had negative coefficients (Table 2).

**TABLE 2:** Five prognostic genes. The coefficient value obtained by LASSO algorithm.

Gene name	Coefficient value
*SALL4*	0.06613418699953
*KRT15*	0.0296694189909953
*OSBPL11*	−0.121246995833747
*SPATA18*	−0.0770127595245775
*TAL2*	−0.18919349247905

**FIGURE 5:**
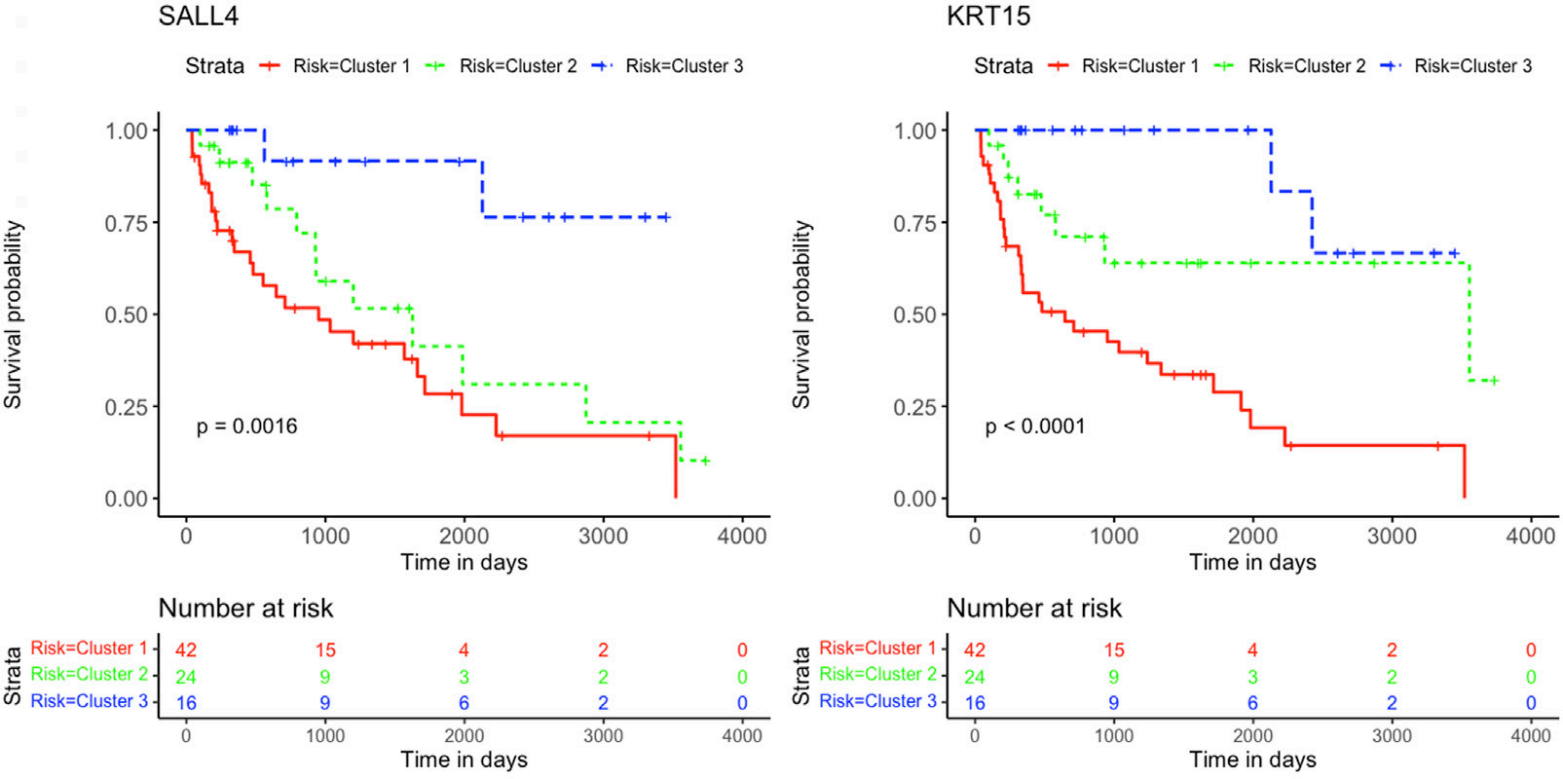
Kaplan-Meier survival curves. Analysis revealed the survival prediction associated with high and low gene expression profiles of *SALL4* and *KRT15* prognostic genes in KIRC patients.

Based on patient statuses, the K-M estimations for overall survival were derived and presented below. The K-M curves illustrate low, intermediated, and high gene expression in blue, green, and red colors, respectively. The K-M curves of genes *SALL4* and *KRT15* with positive coefficient values are presented in Figure 5.

**FIGURE 6:**
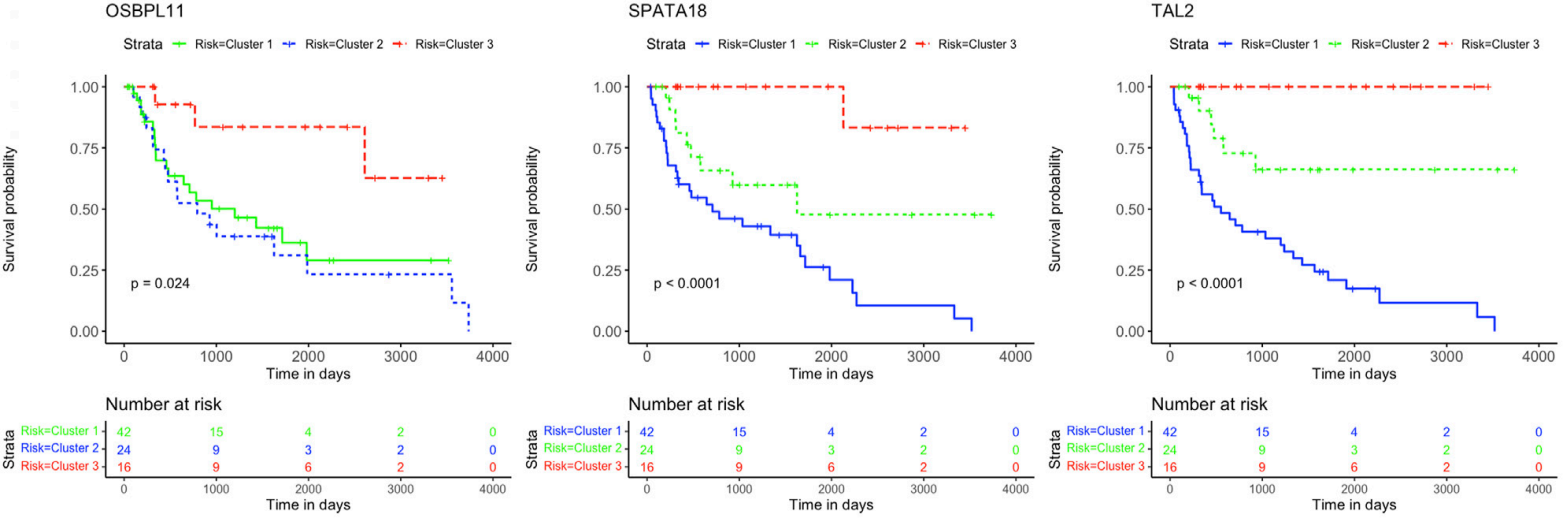
Kaplan-Meier survival curves. Analysis revealed the survival prediction associated with high and low gene expression profiles of *OSBPL11, SPATA18*, and *TAL2* prognostic genes in KIRC patients.

The K-M curves for the three genes *OSBPL11, SPATA18*, and *TAL2* with negative coefficient values are presented in Figure 6.

The five prognostic genes’ estimations and *p*-values in the Cox regression model were all significant, which demonstrates that the altered expression of these genes affects KIRC survival.

### 3.5 Gene expression patterns between risk subcategories

**FIGURE 7:**
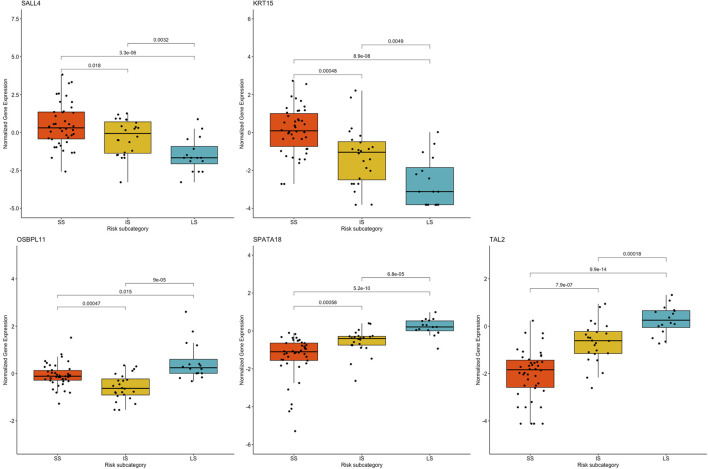
Boxplots based on risk subcategories of the five prognostic genes in KIRC patients. A boxplot was constructed with the normalized gene expression profile of each prognostic gene in all the samples that were categorized into the SS, IS, and LS categories.

One-way ANOVA was performed to assess for differences in the mean normalized gene expression profiles of each of the prognostic genes detected between the risk subcategories. This evaluation included the differences between SS and IS, IS and LS, and SS and LS. Each survival group consisted of a set of samples that make up that risk subcategory, from which a boxplot was created using the normalized gene expression profile of a specific prognostic gene (Figure 7).

All prognostic genes showed a statistically significant difference between SS and LS (*p*-value ≤ 0.015). It is further noteworthy that ANOVA resulted in a statistical difference in the normalized gene expression between IS and LS (*p*-value ≤ 0.0032) as well as between survival IS and SS (*p*-value ≤ 0.018) (Figure 7).

### 3.6 Enrichment analysis

**FIGURE 8:**
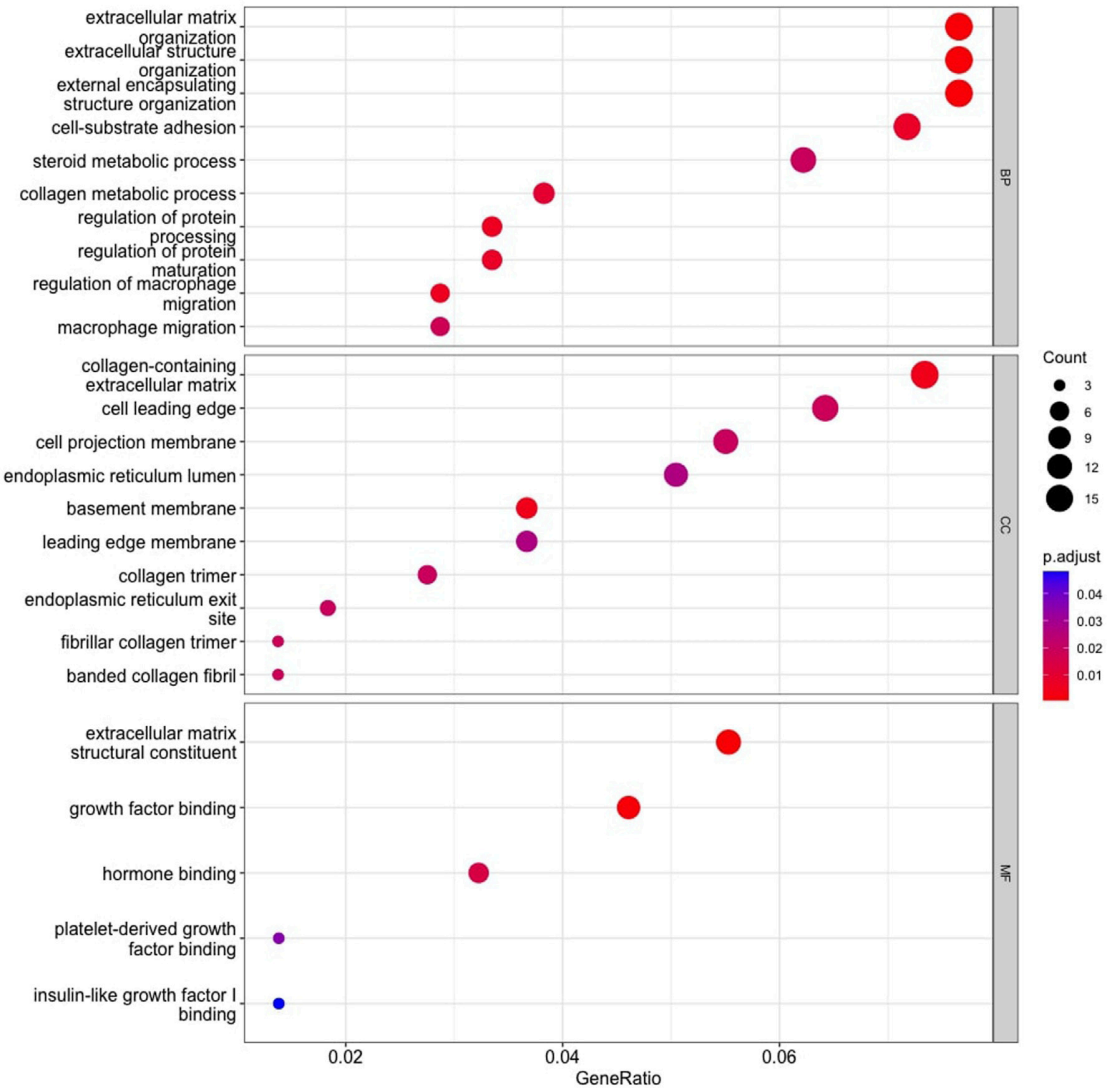
Gene Ontology enrichment analysis. Top 10 functional items of KIRC DEGs based on clusterProfiler. *Functional databases: BP, Biological process; CC, Cellular component; and MF, Molecular function.

**FIGURE 9:**
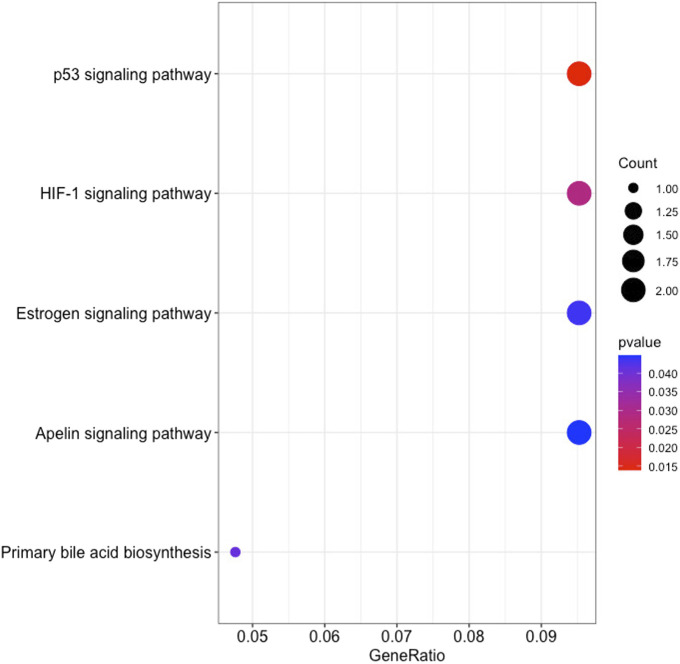
The results of KEGG pathways enrichment analysis of the 48 RFE gene subset based on clusterProfiler.

The GO enrichment analysis illustrated that KIRC DEGs were significantly enriched in biological processes (BP), including extracellular matrix (ECM) organization, extracellular structure organization, and external encapsulating structure organization (Figure 8). In terms of cellular component (CC), collagen-containing ECM, cell leading edge, and cell projection membrane, among other terms were significantly enriched in KIRC DEGs (Figure 8). Lastly, the molecular function (MF), were significantly enriched in ECM structural constituent, growth factor binding, and hormone binding (Figure 8). The KEGG analysis revealed that the 48 gene subset significantly enriched for the p53 signaling pathway, HIF-1 signaling pathway, and estrogen signaling pathway (Figure 9).

## 4 Discussion

The high molecular heterogeneity of RCC necessitates further sub-classification to establish a successful treatment strategy and medical care. Therefore, this study focussed on KIRC as it represents the majority of RCC diagnoses. The study aims to identify subtypes that reflect a genotype-phenotype relationship for KIRC patients that provide a more accurate prognosis, with an emphasis on cancer progression.

This study implemented a normalization method in which the gene expression profiles of eighty-two advanced-stage KIRC samples were normalized with early-stage cancer samples to consider heterogeneity differences in the multi-stage cancer progression. The normalization method corrects for genes that present with high expression variability in early-stage samples but less expression variability in advanced-stage cancer samples. This leads to the availability of more meaningful information to track the cancer progression from early-to advanced-stage, based on the differences in the gene expression profiles.

The normalized gene expression was subjected to a hierarchical clustering method, to detect cancer samples that progress differently in gene expression patterns. The approach allows for the grouping, alternatively, clustering of cancer samples to identify samples within a group/cluster that are similar to each other and different from samples in other groups. This popular method revealed three cancer clusters (subtypes) for KIRC cancer. The three molecularly defined clusters were correlated with the patients’ average OS. It can be noted that patients in Cluster 3 lived on average 657.88 days longer than patients in Cluster 1. Meanwhile patients in Cluster 2 and Cluster 3 live on average 211.95 days and 445.93 days longer than patients in Cluster 1 and Cluster 2, respectively. Thus, the obtained three clusters by the use of our normalization method illustrate different KIRC tumors that progressed differently from early-stage to late-stage cancer development (Figure 3). Consequently, these clusters have different prognoses and can be considered as different subtypes. The results of the hierarchical clustering analysis were subjected to a validation step using an independent GEO dataset (Supplementary Material S1). This test dataset includes sixty-five KIRC samples, and the normalization method also identified three clusters in the GEO KIRC dataset (Supplementary Material S1).

The 48 genes identified through the Machine Learning analysis have the capacity to accurately classify and predict the KIRC subtypes to an extent similar to the use of the 19,556 protein-coding genes. This demonstrates the existence of genetic heterogeneity within KIRC tumors and the ability of our normalization method to recognize this heterogeneity and associate it with prognosis and OS. The gene set contains genes that were reported to play a critical role in the aggressiveness of renal tumors, and our study revealed their involvement in the heterogeneity of the most prevalent and aggressive subtype in renal cancer, KIRC.

Analysis of GO enrichment illustrates the involvement of DEGs in the biological processes that promote tumor aggressiveness. It has been reported that ECM regulates fundamental properties of tumors, such as growth and invasion. The most prevalent genetic mutations in KIRC inactivate the *VHL* gene, which plays a direct role in ECM organization. Therefore, therapeutic approaches to control ECM are currently being investigated and an advanced understanding of KIRC ECM will determine if ECM-modifying drugs are appropriate for KIRC (Oxburgh, 2022). An additional BP enrichment was macrophages that are highly enriched in RCC, and the RCC survival rate is strongly correlated with the inflammatory cytokines secreted by macrophages (Xie et al., 2022).

In terms of the cellular component (CC), KIRC DEGs were significantly enriched in functional elements such as basement membrane (BM). According to a recent study, KIRC is associated with unique basement membrane gene expression patterns, and the characterization of the BM has the potential to guide clinical therapy (Xiong et al., 2022). Cellular component, collagen trimer has been similarly found in studies focused on renal cancer progression (Wang A. et al., 2019), along with molecular function enriched extracellular matrix structural constituent and platelet-derived growth factor binding (Wang A. et al., 2019; van Roeyen et al., 2019). Lastly, MF is significantly enriched for hormone binding, and hormones plays a role in RCC etiology. Hormone receptor expression in RCC cells has been demonstrated to be aberrant (Czarnecka et al., 2016).

Analysis of KEGG pathways revealed signalling pathways that promote cancer progression and resistance to therapies. The *SERPINE1* gene was enriched in the p53 signaling pathway, HIF-1 signaling pathway, and apelin signaling pathway. The interaction between P53 and HIF signaling can promote cancer progression (Zhang et al., 2021). While apelin signaling has also been linked to the development of cancer and its progression (Liu et al., 2021). It is thus noteworthy, that the survival analysis of *SERPINE1* expression in TCGA found a correlation between shorter survival, and the increased tumor grade, lymph node metastasis, and tumor stage (Guo et al., 2023). Therefore, *SERPINE1* plays a crucial role in the progression of KIRC. KIRC patients categorized as SS revealed high levels of *SERPINE1* gene expression, whereas LS displayed low levels of gene expression. Hence, the method tracked the progression of KIRC and further indicated the potential of *SERPINE1* as a therapeutic target for KIRC patients.

Together with *SERPINE1,* the *PGK1* gene was also enriched for HIF-1 signaling pathway. HIF-1 is known to modulate a number of signaling pathways, having a significant impact on the cancer’s response to radiotherapy (Huang and Zhou, 2020). Therefore, a viable approach for sensitization of KIRC to radiotherapy is to target *SERPINE1* and *PGK1.* Also, *PGK1* has been linked to several roles in the development of cancer, tumor progression, and drug resistance. The gene is known to promote sorafenib resistance, which is a first-line treatment for KIRC patients as a tyrosine kinase inhibitor. However, resistance to sorafenib significantly reduces the effectiveness of therapy (He et al., 2022). Therefore, the large patient group (*n* = 42), accounting for about half of the KIRC patients investigated in this study encompassed in SS, may be affected by this resistance to therapy.

Genes *KRT15* and *GPER1* enriched for estrogen signaling pathways can also serve as treatment targets for KIRC patients. Estrogen is known to inhibit the proliferation, migration, and infiltration of RCC cells as well as increase RCC apoptosis (Yu et al., 2013). This study illustrated that the downregulation of *KRT15* had favorable prognostic outcomes for KIRC patients for Cluster 2 and 3 (Figures 5, 7), whereas the downregulation of *GPER1* was linked to unfavorable prognosis in Cluster 1. Therefore, the two genes may serve as valuable prognostic markers for KIRC and a novel developmental approach for enhancing KIRC therapeutics.

This study further identified five prognostic genes as promising prognostic biomarkers and treatment targets for KIRC patients (Table 2). Cox regression together with Kaplan-Meier analyses confirmed the prognostic biomarkers and showed that patients with high levels of *SALL4* and *KRT15* gene expression have a poor survival outcome than patients with low levels of gene expression (Figure 5). While the high gene expression level of *OSBPL11, SPATA18*, and *TAL2* has a favorable survival outcome than patients with a low level of gene expression (Figure 6). Therefore, K-M confirmed that the five genes are effective at diagnosing KIRC patients and predicting prognosis.

The results are supported by previous research, which indicated that the high gene expression level of *SALL4* has a poor survival outcome in comparison to KIRC patients with a low gene expression level (Che et al., 2020). Also, data from Sun et al. (2020) showed that the downregulation of *SALL4* reduces KIRC tumor growth, metastasis, and angiogenesis. Therefore, it is noteworthy that Cluster 2 with intermediate survival followed a similar trend in cumulative survival probabilities as Cluster 1 with short survival (Figure 5). Furthermore, the high gene expression of *KRT15* has also been reported to correlate with a poor prognosis for RCC (Zhang et al., 2023). This study was able to detect *KRT15* as a prognostic gene in the KIRC subtype. The levels of gene expression correspond with the SS, IS, and LS (Figure 7). Previous studies have also reported higher levels of *SPATA18* gene expression associated with favorable OS in the KIRC subtype (Lingui et al., 2023) as well as in RCC (The human protein atlas, 2023a). High expression of *TAL2* has been reported with a favorable OS in RCC (The human protein atlas, 2023b). This is the first article to our knowledge to report *OSBPL11* as a prognostic biomarker. A similar observation as with the *SALL4* K-M curve is observed with the *OSBPL11* gene. The K-M curve of Cluster 2 followed a similar trend in cumulative survival probabilities as Cluster 1 (Figure 6). Therefore, the upregulation of *OSBPL11* could reduce KIRC progression.

ANOVA was used to assess the heterogeneity in the prognostic genes’ mean gene expression profiles, to establish whether SS, IS, and LS samples’ gene expression profiles differ from one another. The prognostic value of the five prognostic genes found was confirmed by ANOVA, which also indicated a statistically significant difference in gene expression between short- and long-term survival. A crucial discovery was made between the gene expression profiles in the intermediate- and long survival as well as intermediate- and short survival. ANOVA showed statistically significant differences between the gene expression profiles of both IS and LS, and IS and SS. This further validates the finding of an intermediate-survival group. The unique gene expression pattern of each of the five prognostic genes were further subjected to a validation step using the independent GEO dataset (Supplementary Material S1). This test dataset verified prognostic genes *OSBPL11* and *TAL2* in the GEO dataset illustrated a similar gene expression pattern for cluster 1 (short survival) and cluster 3 (long survival). The remaining three prognostic genes, *SALL4*, *KRT15*, and *SPATA18* showed similar gene expression patterns for all three clusters (Supplementary Material S1). The five prognostic genes are therefore essential as they may enable an improved KIRC patient prognosis based on the gene expression level of the five genes. Hence, this discovery is important as it is directly correlated with survival and could aid in predicting the outcome of KIRC patients.

The investigation detected molecular mechanisms that allowed for the segregation of three unique cancer clusters (subtypes) that progress differently in gene expression profiles and correlate with KIRC patient survival. Therefore, the normalization method was successfully implemented in this study and hierarchical clustering was able to provide an accurate assessment of the heterogeneity of KIRC. The cellular functions detected by GO enrichment along with the pathogenic genes detected by KEGG pathway analysis further confirmed the contribution to the progression of the disease. Additionally, the heterogeneity of KIRC served as a fuel for therapy resistance and emphasized the urgent need to expand the clinical subtypes for KIRC patients. As a result, this investigation facilitated and contributed to the current KIRC cancer classification with in-depth patient subtyping. The discovery of the five prognostic genes, combined with the biomarkers detected in pathway analysis, can provide a more accurate prognosis, and serve as targets to provide a more effective therapeutic approach for KIRC patients.

## 5 Conclusion

The implemented normalization method has the potential to reveal cancer patients that progress differently (subtypes) and establish a genotype-phenotype relationship between the identified subtypes and the patient’s OS. In this study, correlations between the risk subcategories and gene signatures differentiated short, intermediate, and long survival in KIRC patients. The prognostic capacity of the prognostic genes can successfully classify and predict the prognosis of KIRC patients. Moreover, the prognostic genes were able to segregate patients into additional survival subcategories and thus provide targets that can enhance patient prognosis and aid in the development of individualized treatment approaches.

## Data Availability

The original contributions presented in the study are included in the article/Supplementary Material, further inquiries can be directed to the corresponding author. The source code for the implementation of reproducibility of the analyses for the study is available in GitHub: https://github.com/LiveseyM/KIRC_Subtyping.git.
