# Supplementary material for: Assessment of the progression of kidney renal clear cell carcinoma using transcriptional profiles revealed new cancer subtypes with variable prognosis
Source: Front Genet. 2023 Nov 24;14:1291043. doi: 10.3389/fgene.2023.1291043 (PMC10704507; doi:10.3389/fgene.2023.1291043)
Supplement: Supplementary file 1 [file Table1.DOCX]

Supplementary Material

Assessment of the progression of Kidney renal clear cell carcinoma using transcriptional profiles revealed new cancer subtypes with variable prognosis.

Michelle Livesey, Nasr Eshibona, Hocine Bendou^*^

*** Correspondence:** [hocine.bendou@uct.ac.za](mailto:hocine.bendou@uct.ac.za)

**Supplementary Table S1: List of 48 gene subset selected by RFE.**

| Gene name |
| --- |
| MCUB |
| CD82 |
| IPO11 |
| CPXM1 |
| KDELR3 |
| SALL4 |
| SEC23B |
| PGK1 |
| PDGFRL |
| SERPINE1 |
| GNB3 |
| LPCAT3 |
| CLINT1 |
| IGFBP2 |
| C1orf21 |
| TNFSF18 |
| NLN |
| MMP19 |
| CANX |
| HSD17B4 |
| NACAD |
| ALPK3 |
| ASXL3 |
| FAHD2B |
| OSBPL11 |
| PAQR6 |
| SPATA18 |
| GPER1 |
| DIRAS2 |
| COL22A1 |
| KRT15 |
| CLCN5 |
| NET1 |
| GOLGA6L2 |
| NECTIN3 |
| CPNE7 |
| MCFD2 |
| ZBTB7C |
| WT1 |
| TAL2 |
| CDHR4 |
| FOCAD |
| SLC6A17 |
| ATL1 |
| PCDHA4 |
| PLXNA4 |
| PCDHGC3 |
| CCER2 |
