# Supplementary material for: Assessment of the progression of kidney renal clear cell carcinoma using transcriptional profiles revealed new cancer subtypes with variable prognosis
Source: Front Genet. 2023 Nov 24;14:1291043. doi: 10.3389/fgene.2023.1291043 (PMC10704507; doi:10.3389/fgene.2023.1291043)
Supplement: Supplementary file 2 [file Table2.DOCX]

Supplementary Material

Assessment of the progression of Kidney renal clear cell carcinoma using transcriptional profiles revealed new cancer subtypes with variable prognosis.

Michelle Livesey, Nasr Eshibona, Hocine Bendou^*^

*** Correspondence:** [hocine.bendou@uct.ac.za](mailto:hocine.bendou@uct.ac.za)

The results in the main article were verified using three KIRC-specific GEO datasets; GSE73731, GSE53757, and GSE36895, which includes a total of 70 early-stage and 65 late-stage raw CEL files, which were robust multi-array average (RMA) normalized (Supplementary material Table 1). The GEO dataset was subjected to batch effects removal using ComBat (Supplementary material Figure 1). Our normalization method was then applied to all samples from the three datasets, clustering analysis was performed (Supplementary material Figure 2), and the five prognostic genes identified by TCGA dataset were extracted to create boxplots of the gene expression patterns (Supplementary material Figure 3). The results obtained verified three KIRC subtypes, and two prognostic genes illustrated a similar gene expression pattern to the TCGA dataset for cluster 1 (short survival) and cluster 3 (long survival). The remaining three prognostic genes showed similar gene expression patterns for all three clusters in the TCGA and GEO dataset.

**Table 1: GEO datasets used to verify the results obtained.** An independent test dataset was created from three KIRC-specific GEO datasets.

| GEO datasets | Early-stage | Late-stage |
| --- | --- | --- |
| GSE73731 | 41 | 44 |
| GSE53757 | 24 | 15 |
| GSE36895 | 5 | 6 |
|  | 70 | 65 |

**Figure 1: PCA plots before and after batch effect removal.** The three GEO datasets were subjected to batch effect removal using ComBat. The GEO expression dataset after batch effect removal were used for further analysis.


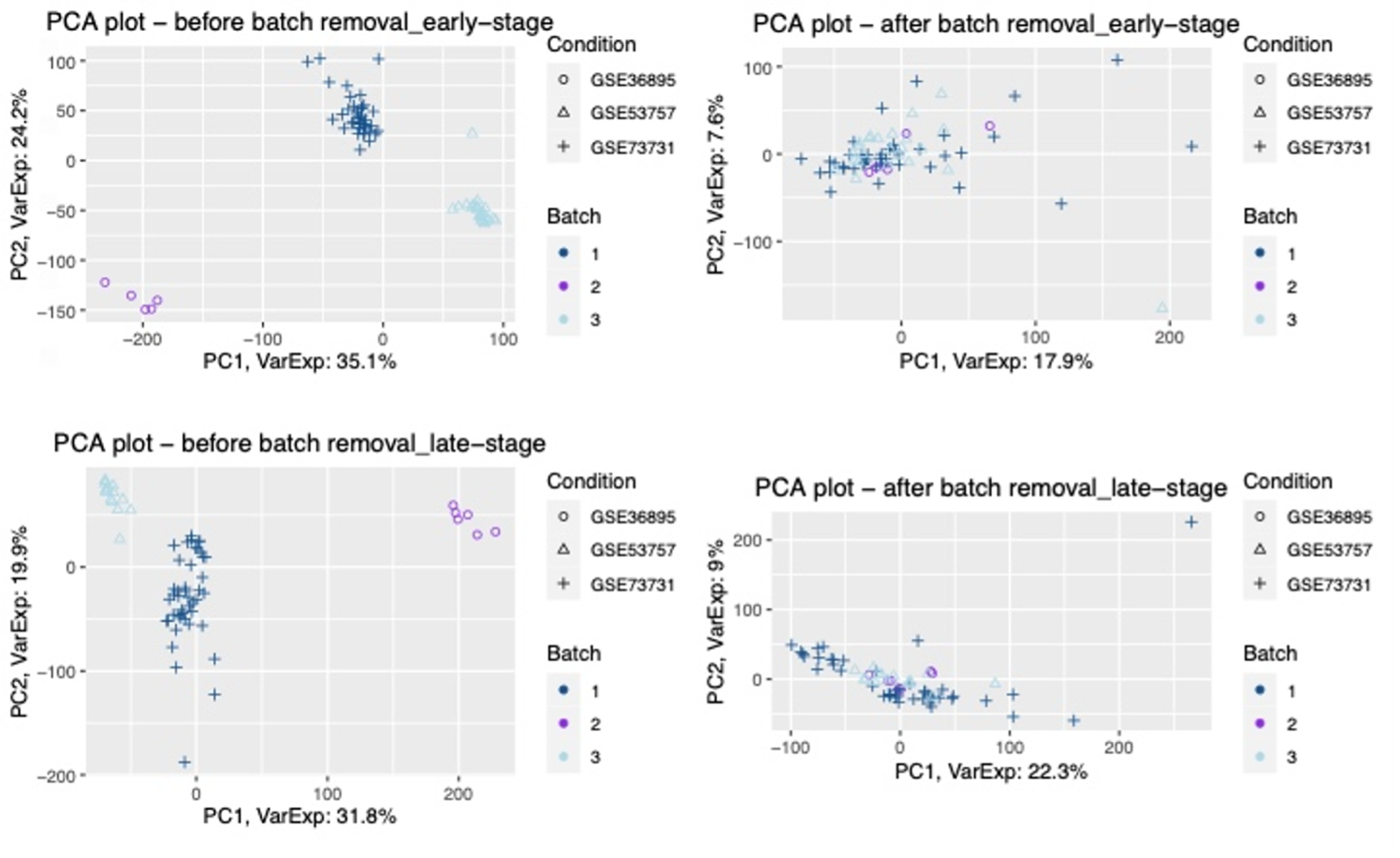


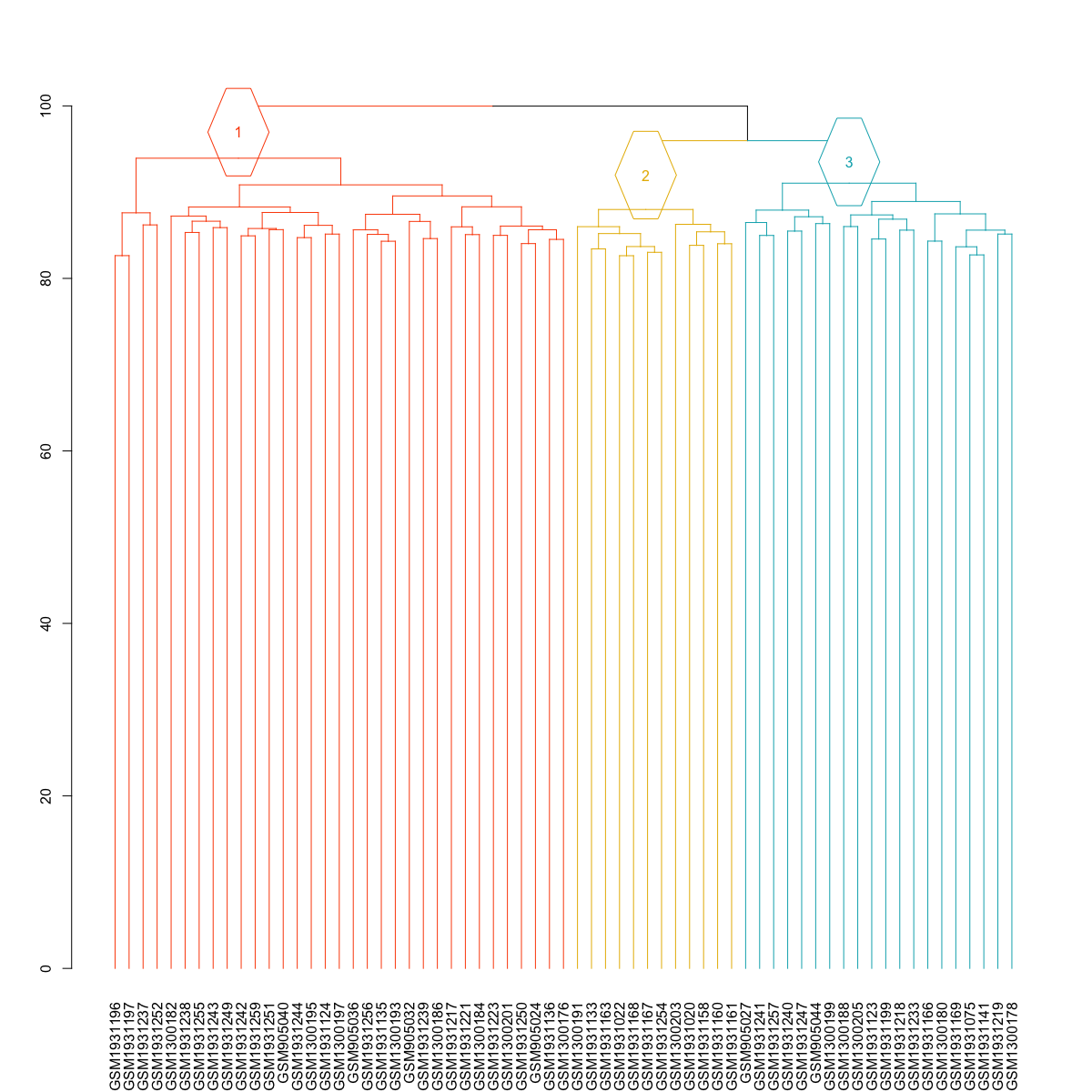
**Figure 2: Hierarchical clustering dendrogram of KIRC patients in GEO dataset.** The normalized gene expression of the sixty-five KIRC cancer samples were subjected to clustering analysis, to reveal the grouping of cancer samples. The GEO dataset verified the three KIRC subtypes.

**
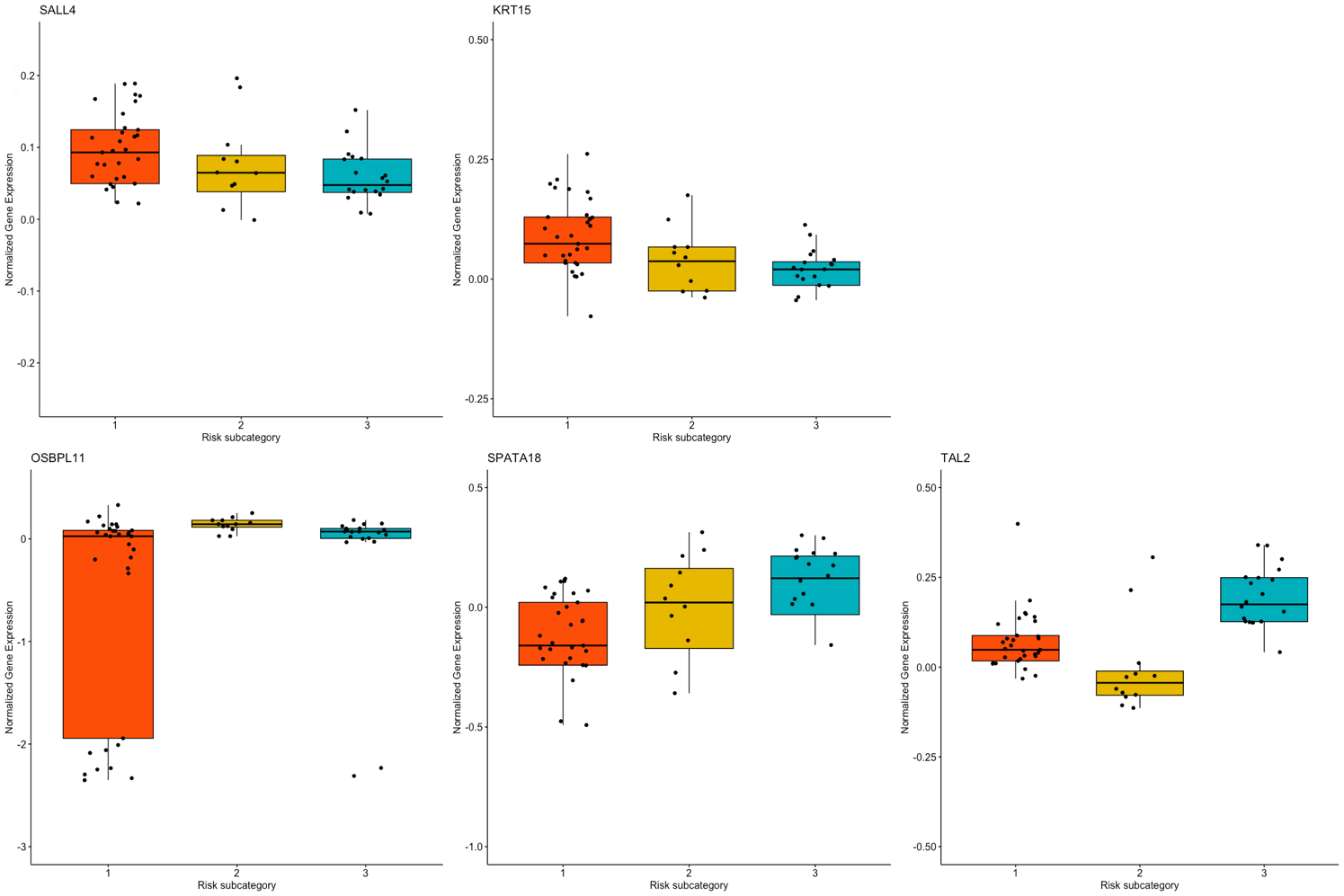
Figure 3: Boxplots were constructed of the five prognostic genes identified by the TCGA dataset.** The normalized gene expression profiles of the five prognostic genes in all the samples that were categorized into clusters was extracted from the GEO dataset. Genes *OSBPL11* and *TAL2* in the GEO dataset illustrated a similar gene expression pattern to the TCGA dataset for cluster 1 (short survival) and cluster 3 (long survival). The remaining three prognostic genes, *SALL4*, *KRT15*, and *SPATA18* showed similar gene expression patterns for all three clusters in the TCGA and GEO datasets.
